# Mixed lymphocyte reaction induced by multiple alloantigens and the role for IL-10 in proliferation inhibition

**DOI:** 10.4103/2321-3868.126088

**Published:** 2014-01-26

**Authors:** Junyi Zhou, Weifeng He, Gaoxing Luo, Jun Wu

**Affiliations:** 1 State Key Laboratory of Trauma, Burn and Combined Injury, Institute of Burn Research, Southwest Hospital, the Third Military Medical University, China; 2 Chongqing Key Laboratory for Proteomics of Diseases, Chongqing, China; 3 State Key Laboratory of Trauma, Burn and Combined Injury, Institute of Burn Research, Southwest Hospital, The Third Military Medical University, Chongqing Key Laboratory for Proteomics of Diseases, Chongqing, 400038 China

**Keywords:** Interleukin-10, T-lymphocytes, intraclonal competition, mixed lymphocyte reaction

## Abstract

The frequency of T cells that can respond to alloantigens is unusually high. It remains unclear how T cells would respond when stimulated by multiple major histocompatibility complex (MHC) disparate alloantigens in the same cultures. In this report, we examined potential interactions of T cell clones that were stimulated simultaneously by two sets of complete MHC disparate alloantigens using mixed lymphocyte reaction (MLR). In this assay, we observed that proliferation of B6 lymphocytes (H-2b) stimulated by both BALB/c (H-2d) and C3H (H-2k) allogeneic cells was not increased but rather reduced as compared to B6 cells stimulated with either BALB/c or C3H allogeneic cells. Interestingly, interleukin (IL)-10 expressions at both protein level and mRNA level was significantly increased in cultures stimulated with the two MHC alloantigens, while IL-2, tumor necrosis factor (TNF)-α, transforming growth factor (TGF)-β1 production did not show any differences. In addition, Foxp3 mRNA expression was comparable amongst all groups. In conclusion, we observed an inhibitory effect in T cell proliferation in response to multiple MHC mismatched alloantigens in MLR, and this effect might be associated with the upregulation of IL-10 expression.

## Introduction

Interactions among different clones of CD4^+^ and CD8^+^ T cells are found in many immune responses, including T cell development, maturation, reconstitution, antigen recognition and different stages of T cell activation, and such interactions play an important role in the sustaining of immunologic homeostasis.[1,2] Although co-operation and competition between different clones of T cells with the same epitope specificity has been well studied. The possibility that competition may exist among T cells with different antigen specificities in their immune responses is rooted in transplantation and has only been recently explored.[3,4] The interactions between different clones of T cells and their influence on immune reactions were identified by evaluating immune rejection in response to different major histocompatibility complex (MHC) molecules, minor histocompatibility antigens or histologic specific antigens following organ or tissue transplantation.[5] The report by Lee *et al.*, describing the low immune response to grafts with more complicated antigen compositions further indicated that intraclonal competition might reduce the total strength of the immune reaction, and this effect may be used to induce graft-specific tolerance.[6] However, the direct evidence for intraclonal competition between T cells of different antigen specificities remains limited, and the mechanism has not been fully elucidated.

**Table 1:** 

Access this article online	
**Quick Response Code:**	**Website:** www.burnstrauma.com
	**DOI:** 10.4103/2321-3868.126088

The mixed lymphocyte reaction (MLR) is a traditional tool in the area of cellular immunology. With the understanding of how the recognition of the MHC-antigen complex by the T-cell receptor (TCR) occurs, it became clear that the basis of T-cell alloreactivity in MLR was essentially similar to the recognition of the nominal peptide epitopes presented by self-MHC molecules.[7] Thus, the MLR provides a simple and efficient *in vitro* model for the study of T-cell activation and proliferation.

In this study, we investigated the proliferation of T cells upon stimulation with two different alloantigens (MHC H-2d and H-2k) in the MLR model. In this setting, we assessed competition between two populations of T cells reactive to two sets of different allogeneic antigens. The lymphocytes stimulated with two different allogeneic antigens showed decreased proliferation compared to the groups triggered by a single antigen, which suggested a possibility that competition between the two populations of lymphocytes could inhibit the proliferation of both populations. The mRNA expression levels and the secretion of interleukin (IL)-10 were significantly increased in the lymphocytes that were exposed to both of the MHC antigens, thereby indicating that the upregulation of IL-10 might play a role in this competitive suppression effect.

## Materials and methods

### Animals

Eight- to ten-week-old inbred BALB/c (H-2d), C57B/L (H-2b) and C3H (H-2k) mice were purchased from the Chinese Academy of Sciences, Shanghai Laboratory Animal Center. The mice were then bred in a specific pathogen-free unit.

### Mixed lymphocyte reaction

Spleen lymphocytes were harvested from C57BL/6 mice and were used as responder cells. The spleen lymphocytes from either BALB/c mice or C3H mice were pretreated with mitomycin C (MMC, 25 µg/mL, Hyowa Hakko Kogyo BioCo., Tokyo, JP) and were used as stimulator cells. The MLR was performed by seeding 1 × 10^5^ responder cells into the wells of a round-profile 96-well plate, and 1 × 10^5^ MMC-treated allogeneic cells from BALB/c mice, C3H mice or 1:1 mixed allogeneic cells from both BALB/c and C3H mice were added to each well in a final volume of 200 µl of lymphocyte medium. Every group set three repetitions.

### Lymphocyte proliferation assays

Lymphocyte proliferation was assessed using a CCK-8 cell counting kit (DOJINDO, JP).[8] A CCK-8 solution was added to each well of the MLR, and was incubated for 4 h before measuring the OD values in a microplate reader at 450 nm. The stimulation index (S.I.) was calculated as follows: S.I.= OD of responder cells in wells with stimulator cells added/OD of the same responders in wells containing responder cells only.

### Cytokine secretion assays

The secretion of IL-2 and IL-10, tumor necrosis factor (TNF)-α and transforming growth factor (TGF)-β1 was measured by ELISA.[9] The culture medium supernatant was collected after 72 h of MLR culture. Aliquots of 20 µl of supernatant from all the groups were added to ELISA microplates (R&D Systems, MN). Each group was assayed in triplicate. The absorbance OD was measured at 405 nm with a microplate reader (Bio-Rad, CA).

### Expression of IL-10 and Foxp3 mRNA

After the MLR had proceeded for 72 h, the co-cultured spleen mononuclear cells were collected, and total RNA was prepared with an RNA extraction kit (Qiagen Co., Shanghai). The following PCR primers were used to amplify IL-10 mRNA: F, 5′-tac agc cgg gaa gac aat aac t-3′ and R, 5′-aca ccc agg aaa gac agc a-3′. The following primers were used to amplify Foxp3: F, 5′-aca ccc agg aaa gac agc a-3′ and R, 5′-aca ccc agg aaa gac agc a-3′. The relative quantity was analyzed by real-time PCR (ABI, JP).

### Statistical analysis

All data are presented as means ±S.E. The statistical analysis of measurement data was performed by one way ANOVA with Statistical Package for the Social Sciences software (SPSS v13.0). Differences with *P* < 0.05 were considered significant.

## Results

### Proliferation inhibition of T cells stimulated by two sets of alloantigens

**Figure 1:**
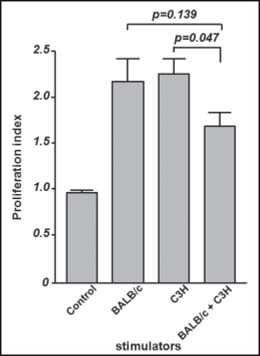
Proliferation response of the lymphocytes isolated from C57BL/6 mice to different stimulators. The data are expressed as stimulus index (S.I.). In contrast to the single type allogeneic cell stimulated groups, the lymphocytes which encountered with two types of allogeneic cells exhibited a mild reaction to the stimulators (*P* = 0.047 *vs*. stimulators from C3H alone, and 0.139 *vs*. stimulators from BALB/c alone).

In the MLR reaction, spleen lymphocytes from C57/BL mice showed a strong proliferative response when stimulated with lymphocytes from both BALB/c and C3H mice. When the two types of stimulating cells were mixed at a 1:1 ratio and were then added to the responder cells, the expansion of C57/BL lymphocytes was not enhanced. Rather, the proliferation index of the group was slightly decreased (*vs*. C3H alone and BALB/c alone, *P* = 0.047 and 0.139, respectively), indicating that a 1:1 mixture of the stimulating cells had a suppressive effect [Figure 1].

### Increased IL-10 secretion by T cells stimulated by two sets of alloantigens

**Figure 2:**
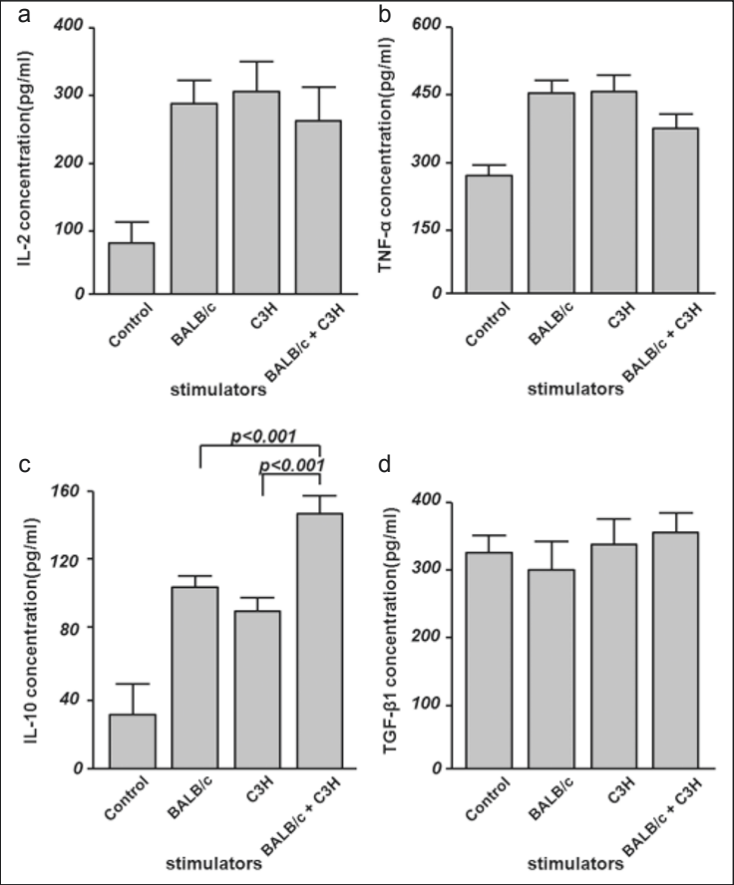
Cytokine secretion of the lymphocytes in mixed lymphocyte reaction (MLR). (a, b and d) No difference was detected in the concentration of IL-2, TNF-α and TGF-β1 among all wells of lymphocytes, which were activated by either one or two types of allogeneic stimulators.(c) Lymphocytes activated by both types of MHC antigen showed significantly higher level of IL-10 secretion than that of the lymphocytes stimulated any one of the MHC antigen.

Cytokine secretion was determined by enzyme-linked immune (ELISA) after 72 h of MLR. The secretion of IL-10 was significantly increased in lymphocytes that were activated by both types of allogeneic stimulating cells when compared to any single MHC antigen-stimulated group (*P* < 0.001) or the resting controls. However, no differences were observed in the concentrations of the immune active cytokines IL-2 and TNF-α or in the level of the immune suppressive cytokine TGF-β1 in the culture medium supernatant [Figure 2].

### Upregulation of IL-10 gene expression

**Figure 3:**
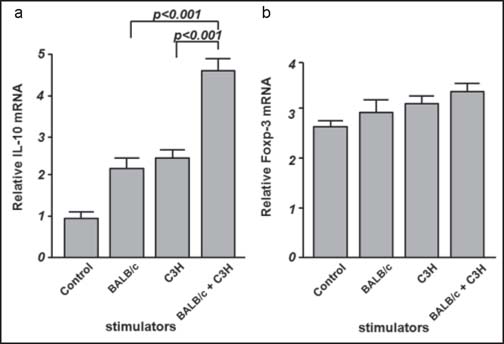
Expression of IL-10 and Foxp3 mRNA in spleen mononuclear cells in MLR. (a) Co-instantaneous stimulation with both types of allogeneic cells induced a significantly increased IL-10 expression when compared to single type allogeneic stimulators (*P <* 0.001). (b) No difference was detected in the expression of Foxp3 mRNA among all reaction groups or resting control.

The upregulation of IL-10 was observed in all groups as a negative feedback of the proliferation reaction. Furthermore, the expression of IL-10 mRNA was also increased in the two allogeneic MHC-stimulated groups compared to the single stimulator groups (*P* < 0.001). However, the expression Foxp3, a marker of regulatory T cells (Treg), showed no difference among the groups [Figure 3b].

## Discussion

Competition among T cell clones was first observed while studying access to antigen bearing antigen presenting cells (APCs). TCRs with higher affinity had an activation advantage, and affinity competition was further observed during the development and maturation of lymphocytes. [9,10] While mounting evidence suggests that in addition to competition between T-cell clones for the same epitope, T cells with distinct antigen specificity may compete with each other when encountering different antigens at the same time. In a transplantation immune reaction, the response to one mH antigen may dominate the response to others. [11] Lee *et al.*, reported that the rejection of a composited tissue allograft was lower than the rejection of grafts of any tissue component alone.[6] These phenomena suggest that competition between lymphocytes with different antigen specificities may reduce the total strength of the immune reaction, thus raising the possibility of a new approach for tolerance induction following transplantation.

MLR is a traditional *in vitro* model to assess antigen recognition and the activation and proliferation of T lymphocytes. Thus, examining the kinetic parameters of a MLR may provide evidence of the mechanisms responsible for T-cell activation *in vivo*. The affinity competition of T cells has been successfully exhibited in MLR, confirming it as a viable model for the study of intraclonal interactions.[12] Therefore, splenocytes with two different MHC phenotypes were used in the MLR as stimulators to observe the competition between the two groups of T lymphocytes that were reactive to these two antigens.

A repertoire of 0.5% to 2.0% cell clones in the T cell repertoire could be stimulated to enter proliferation by the MHC disparate stimulators in MLR.[13] Therefore, if these cells were stimulated with splenocytes possessing two different MHC phenotypes, more T-cell clones should be activated. However, in our model, the proliferation reaction was not enhanced in this situation. In contrast, the proliferation of these T lymphocytes was slightly reduced. Thus, we speculated that intraclonal competition between the two populations of T cells might be occurring in the reaction.

Although the interaction between T cells with same epitope specificity has been finely elucidated and is closely related to TCR affinity competition and the activation of affinity-dependent IL-2 signaling, the mechanism of intraclonal competition between T cells with different antigen specificities remains unclear.[14] Co-stimulating signal expression, cytokine response and the induction of regulatory T cells might be involved in these polyclonal interactions.[15]

IL-2 is one of the early cytokines in immune activation, and therefore is a sensitive measure of T-cell proliferation and differentiation.[16] Competition for IL-2 is involved in the intraclonal competition between CD4^+^ T cells with same antigen specificity.[17,18] However, Taams *et al.*, reported that the suppressive effect of polyclonal T cell responses was not due to passive IL-2 consumption or to T-cell cytolysis.[19] We also confirmed that the concentration of immune active cytokines such as IL-2 and TNF-α was equivalent among all MLR groups, thus the consumption of immune active cytokines is unlikely to be the mechanism of suppression in the clonal competition observed in this model.

In our experiments, the immune suppressive cytokine lL-10 was significantly upregulated at both protein level and mRNA level in the two antigen-stimulated group, suggesting a possibility that this proliferation inhibition in intraclonal competition is related to the upregulation of IL-10 in lymphocytes.

As a negative feedback regulation, activated Th cells secret large amounts of IL-10 to suppress the proliferation of T cells and to control the total strength of immune reaction.[20] In this model, although the exact ratio and phenotype of the Th cells that secreted IL-10 in the MLR was not clear, there may be more Th cells activated by the simultaneous presence of two different antigens that could mediate the suppression of the primary T-cell reaction.

Another subset of IL-10-expressing lymphocytes is the CD4^+^ Foxp3^+^ Tregs, a group of T lymphocytes with strong immune regulatory functions.[21] However, the IL-10 expression of Tregs is mediated by TGF-β1, and natural Tregs promote the development of IL-10-producing Tregs from naive T cells.[22] However, neither TGF-β1 secretion nor Foxp3 expression showed an increase in the lymphocytes stimulated with two antigens. Although the role of the Tregs in lymphocyte intraclonal competition remains controversial, the Foxp3^+^ Tregs did not appear to participate in proliferation inhibition in this model.[23]

In conclusion, intraclonal suppression of T-cell proliferation was observed in the MLR in response to two sets of MHC incompatible allogeneic antigens. And this intraclonal competition may be related to the induction of IL-10 secretion. However, the exact mechanism remains to be determined.
